# Highlighting of Urinary Sodium and Potassium among Indonesian Schoolchildren Aged 9–12 Years: The Contribution of School Food

**DOI:** 10.1155/2019/1028672

**Published:** 2019-04-03

**Authors:** Farapti Farapti, Muji Sulistyowati, Kurnia Dwi Artanti, Stefania Widya Setyaningtyas, Sri Sumarmi, Bibit Mulyana

**Affiliations:** ^1^Department of Nutrition, Faculty of Public Health, Universitas Airlangga, Surabaya 60115, Indonesia; ^2^Department of Health Promotion and Behavior Science, Faculty of Public Health, Universitas Airlangga, Surabaya 60115, Indonesia; ^3^Department of Epidemiology, Faculty of Public Health, Universitas Airlangga, Surabaya 60115, Indonesia; ^4^Doctoral Program at Faculty of Psychology, Universitas Airlangga, Surabaya, Indonesia

## Abstract

**Background:**

Sodium (Na) and potassium (K), the essential nutrients, have vital role in promoting cellular growth including growth and development of children. Excessive Na intake and inadequate K consumption, which consequently increases the risk of cardiovascular disease, have been reported. Spot electrolyte urine was highly correlated and validated with gold standard to estimate electrolyte dietary intake. This study aimed at predicting sodium and potassium intake using morning spot urine among Indonesian schoolchildren.

**Methods:**

A cross-sectional study was carried out in 155 healthy elementary students aged 9–12 years. Spot urine samples were collected and analyzed for Na, K, and creatinine. Predicted 24 h Na and K excretions were compared to the Indonesian recommendation dietary allowances. The Na and K contribution from school food was reported by observing directly and the dietary recall method.

**Results:**

A total of 80 boys and 75 girls recruited as samples in this study demonstrated that their estimated urinary Na and K were 105.42 ± 66.05 mmol/day and 16.39 ± 12.57 mmol/day, respectively. Na intake was on average higher than recommended; meanwhile, almost all subjects showed very low compliance of K intake recommendation. Furthermore, food intake at school contributed to those conditions. Na and K content of school food contributed 33% and 29% of the daily intake of each nutrient and contributed 125% and 25% higher than the Na and K school standard, respectively.

**Conclusions:**

Indonesian schoolchildren aged 9–12 years are categorized by excessive Na intake and very deficient K intake. The present study highlights the need for policies in the environmental school setting to reduce Na intake and K intake.

## 1. Introduction

Sodium (Na) and potassium (K) are two essential nutrients having an important role in normal bodily functioning. Na and K are two principal electrolytes in extracellular fluid and intracellular fluid, respectively, which together have a vital role in regulating body fluids, maintaining osmotic equilibrium, stabilizing acid-base balance, determining membrane potentials of smooth muscles and nerves, regulating molecules to transport actively across cell membranes, and promoting cellular growth [1]. It is widely known that excessive sodium consumption and deficit of potassium intake have an important role in the pathogenesis of hypertension and more strongly associated with blood pressure than either Na or K alone [2, 3]. Furthermore, epidemiology studies have revealed that high Na and low K intake alone and together have risk for adverse health effects such as cardiovascular diseases [4, 5], stroke [6], chronic kidney disease [7], obesity [8], and all-cause mortality [9].

Population studies reported that most population around the world consume less than the recommended intake of K; however, unfavorably high Na intakes remain prevalent around the world. The part played by these nutrients has been thoroughly studied in adults and children, but most studies have been concerned with adults [10, 11]. The studies on the effect of Na and K directly on children were few; a previous study reported that Na and K were associated with hypertension and obesity among children [12]. A prospective study showed people with excess salt intake and hypertension at an early age are more likely to develop hypertension, cardiovascular disease, and stroke in the future [13].

Since dietary habits established in childhood can generally be tracked into adulthood and the risk of many noncommunicable diseases is closely related with dietary intakes, concerning dietary habits at early age is very crucial to prevent future diseases and increase beneficial effects on human health [13, 14]. Recent studies described unhealthy dietary intakes are common among children; it was related to low nutrition knowledge, preference, and bad dietary habits in children [15, 16]. The nutrients, for instance, Na and K intake, are closely related with dietary habits in children. Sodium is mostly used in the form of sodium chloride (salt), and it is the widely used ingredient in the processed food. The addition of salt to food will improve the flavor, which also increases saltiness and suppresses bitterness. A previous study revealed that children prefer higher salt taste than adults do and the addition of salt to foods increases children's consumption of those foods and causes excessive sodium consumption [17]. On the other hand, deficit potassium intakes in children have been reported by several researchers from many countries. Although diets containing abundant fruits and vegetables are potassium rich, it was known that children consume fruits and vegetables less than 20% of recommended [18].

Twenty-four-hour urine collection is gold standard to assess dietary Na and K; however, this method is difficult to perform and not convenient in studies involving children [19]. Spot urine Na and K are well documented and proven accurately in predicting 24 h urinary Na and K levels among adults and children [20, 21]. In Indonesia, most studies assessing Na and K intake were performed with dietary assessment methods; no data were available for obtaining these nutrients in children by urinary excretion. Since dietary Na and K can be a useful marker and predictor of dietary quality related to human health and because of limited scientific data supporting, it is important to examine Na and K intake among young population.

Therefore, the study aimed at assessing and evaluating urinary Na and K using morning spot urine among Indonesian subjects aged 9–12 years with the focus on evaluating dietary Na and K in children on the day of the school since schoolchildren spent one-third of their time at the school.

## 2. Materials and Methods

This cross-sectional study, which was conducted between April and October 2018, enrolled the students from elementary school (9- to 12-year-olds) in Indonesia. We studied 155 Indonesian subjects from the school at Surabaya, one of the metropolitan areas in Indonesia. To accomplish this project, children attending the 4th and 5th grade were recruited as subjects in this study after getting permission from the headmaster. Prior to data collection, all students had been explained and given written information about informed consent on the project, and then it had been granted to children to get parental permission and signature; thus, written consent was obtained from the child, as well as the child's parent. Informed consent was arranged in accordance with the ethical standards laid down in the Declaration of Helsinki, and the Ethical Committee of Faculty of Public Health, Universitas Airlangga, approved the protocol of this study with the ethical number 446-KEPK.

### 2.1. Data Collection and Demographic Characteristics

Data collection was done through structured interviews, anthropometric measures, questionnaires, collection of spot morning urine sample, and 2 × 24 h dietary recall by trained researchers. Sociodemographic characteristics, namely, age, sex, class grade, and pocket money, were obtained by trained researchers using a structured questionnaire. A research team supported children to complete urine collection and to collect anthropometric measures.

### 2.2. Anthropometric Measures

The collection of anthropometric data including height and weight was measured using standard protocols (wearing light clothing, with shoes removed) by trained researchers. Body weight measurement was obtained by using an electronic scale (TANITA® TBF-300A, capacity 200 kg, accuracy 100 g), and the height was obtained using a stadiometer (capacity 200 cm, accuracy 1 mmol). Body mass index (BMI) was computed as kg/m^2^. Body mass index (BMI) values were converted to age-adjusted and sex-adjusted BMI *z*-scores and then it was plotted on the WHO BMI-for-age growth charts and obtained a percentile ranking, classifying children as underweight (less than the 3rd percentile), normal weight (3rd to less than the 85th percentile), overweight (85th to less than the 97th percentile), or obese (equal to or greater than the 97th percentile) [22].

### 2.3. Dietary Intake and School Food

A nutritionist interviewed and recorded dietary energy and macronutrient intake of subjects by asking subjects to recall a two-day food intake during a week of data collection. Participants were observed twice at mealtime on the same day (weekday) of recalling food intake. The food items consumed on the day of the school were recorded including home-packed lunch, school lunch meal, and canteen foods. Portion and sizes of school food were estimated using household measures. The nutritionist clarified the accuracy of data by clarifying menu, portion, and size of the foods consumed by the subjects using food model and Indonesian food book [22, 23]. The contribution percentage of nutrient intake on the school food (energy, sodium, and potassium) was calculated by dividing those dietary intakes while at the school by those daily intakes. The requirement percentage of nutrient intake on the school food was obtained by dividing those dietary intakes while at school by those based on school food standard.

### 2.4. Urinary Sodium, Potassium, and Creatinine Excretion

The Na and K intake in this study was estimated based on urinary Na and K excretion. The day before urine collection, all participants were given written and verbal instructions on how to collect spot urine correctly. The first urine of the day was collected in the bottles provided by the researcher. Then, the pupils brought the urine bottle to school and gave it to the researcher team to be delivered at laboratory. Urinary Na and K concentration was assessed by the indirect ion-selective electrode method [24], whereas urinary creatinine was assessed using the Jaffe reaction [25].

### 2.5. Estimation of 24 h Sodium, Potassium, and Creatinine

Analysis of urine was performed on the first morning sample. Estimation of the daily urinary electrolyte excretion to creatinine (Cr) ratio in spot urine samples was reported to depend on the accuracy of predicting urinary 24 h Cr calculated based on the data by Remer et al. [26]. Urinary Na/Cr or K/Cr was calculated as follows: (urine Na or K in mmol/L)/(urine creatinine in mg/dl) *×* 0.0883.

The molecular weights of Na (23 g/mol) and K (39 g/mol) were used to convert millimoles of Na and K, respectively, to milligrams. The results of urinary Na and K excretion data are presented as mmol/day and mg/day as well as the salt equivalent. Salt equivalents were calculated by dividing Na concentration in mg by 390.

### 2.6. Statistical Analysis

Statistical analysis was conducted using SPSS 21 (IBM SPSS), and two-sided *p* values less than 0.05 were considered statistically significant. The Kolmogorov–Smirnov test was performed to test variables for normality. Descriptive statistics were used to describe participant characteristics. Continuous variables were summarized as mean and standard deviation, and categorical variables were presented as counts/numbers and percentages. Independent samples *t*-test and the Mann–Whitney test were used to identify sex differences for urinary electrolyte excretion. The one-way ANOVA test and post hoc test were used to analyze factors contributing estimated Na and K intake.

## 3. Results

A cross-sectional study was performed between April and October 2018. A total of 155 healthy primary students (9–12 years) were randomly selected using a multistage cluster sampling procedure from approximately 305 pupils from the 4th and 5th grade at Surabaya elementary school in Indonesia. A total of 80 boys and 75 girls were recruited as samples in this study. From Table 1, it can be seen that the average age was 10.15 ± 0.77 years and the mean body mass index was 19.53 ± 4.4 kg/m^2^ with 23.9% of subjects categorized as obese. The median daily pocket money was Rp10000 with a mean of Rp11954.84 ± 5.197.83.

Table 2 demonstrates that all variables of urinary excretion were similar in boys and girls (*p* > 0.05). The estimated urinary Na or predicted Na intake was 105.42 ± 66.05 mmol/day, equivalent to 6.17 g/d of salt. By contrast, mean urinary K excretion was 16.39 ± 12.57 mmol/day or 639.15 ± 490.04 mg/d; the estimated average daily K intakes for boys and girls were 17.37 mmol and 15.34 mmol, respectively. Consequently, the mean of the Na/K ratio was 7.71 ± 4.02 (mmol) or 4.55 ± 2.37 (mg).

Seventy-four percent of children had Na consumption higher than 1500 mg/d, and 41.3% of subjects consumed Na intake more than 2300 mg/d. For estimated K intake, only 11.6% and 3.9% of the subjects consumed K intake higher than 1000 mg/d and 2000 mg/d, respectively (Table 3).

The data of dietary intake (energy, Na, and K) from the dietary method were presented as mean and standard deviation in Table 4. It can be seen that the total energy intake of subjects was 1896.55 ± 276.4 kcal/d, and 42.76% was obtained from the food on the day at school. The Na content of the school food was 606.9 ± 218.9 mg and contributed nearly one-third percent from daily Na intake. On the contrary, the K content of the school food contributed only 29.78% compared to daily intake, and these intakes contribute only a quarter percent based on nutritional school standard.

Table 5 gives a description of factors contributing estimated Na and K intake. For Na intake, there were no significant differences in age, amount of pocket money, and energy in full day; however, BMI *z*-score and body weight were significantly associated with estimated Na intake. Furthermore, it is observed from the post hoc test that compared to group 1 (<1500 mg/d) energy of school food was significantly higher in group 3 (2300 mg/d) with *p* value 0.04. For K intake, the median value of 500 mg/d was categorized as group 1 and higher than 1000 mg/d as group 3. As can be seen, BMI *z*-score, body weight, and energy of school food correlated significantly with estimated K intake.

## 4. Discussion

Sodium (Na) and potassium (K) have been the main topics in recent years since these nutrients have been closely related to the risk of many noncommunicable diseases in adults and children [9, 13]. Moreover, in the industrialized nations where the majority intake comes from processed food and commercially packaged foods, it is globally known that most people have excessive Na (salt) consumption and deficit of K intake. Recently, it was found that high Na (salt) and or low-potassium diet become public health problem in the worldwide [9–11].

Assessing dietary Na and K by using urinary excretion had been performed in several countries including Indonesia [27]. However, according to our best knowledge, the present study is the first Indonesian study using urinary electrolyte excretion to assess these nutrients in children aged 9–12 years since there are no scientific data to explain this condition in Indonesian children. Our study involved children subjects aged 9–12 years at elementary school. Assuming that children in that age can cooperate well and answer clearly, they were asked to complete urine collection by themselves and recall of food intake. The previous studies revealed the school children aged 9–12 years were categorized as pre-early adolescents, and it is reasonably narrow that they can think logically and be aware of and responsible for their food choices [17, 28].

In our study, predicted 24 h Na excretions were assumed to reflect 24 h Na intakes. This study with a total of 80 boys and 75 girls recruited as samples demonstrated that predicted Na intake was 105.42 ± 66.05 mmol/day or 2424.77 ± 1519.09 mg/d, equivalent to 6.17 g/d of salt. These findings are similar to those observed by previous studies. Service et al. [29] in 168 children aged 9–12 years reported Na intake of approximately 110 ± 53 mmol/d; Grimes et al. observed 103 ± 43 mmol/d [30] in 193 Australian subjects aged 5–13 years, and 113 ± 3 mmol/d intake of Australian salt and other nutrients was reported in the children (SONIC) study among 383 children aged 9–12 years [31]. On the other hand, several authors have reported higher intakes: 132.7 ± 51.4 mmol/d reported by Aparicio et al. [32] in 205 Spanish children aged 7–11 years and the 129 mmol/d among boys and 117 mmol/d among girls from Italian children with a mean age of 10.1 ± 2.9 years [12]. Mean of urinary Na intake less than 100 mmol/d was reported in the previous studies. 96.57 ± 61.67 mmol/d was reported by El Mallah et al. among 1403 Lebanese children aged 6–10 years [34]. 97.19 mmol/day among Moroccan children aged 6–18 years [35] and 80.7 ± 3.4 mmol/d in 111 British (South London) subjects aged 8–9 years were observed by Marrero et al. [36], and the median of 94.4 and 95.0 mmol/24h was demonstrated by Libuda et al. [37] in a group of German boys and girls aged 10–13 years, respectively. Although the intake was lower than that in our findings, it is still higher than maximum RDA. Consequently, Na intake is still a public health problem worldwide [11].

Assuming that the Na eliminated in urine arose from the diet, the Na intake in our study was classified as high (161.65%) compared to Indonesian RDA for children aged 9–12 years (1500 mg/d) [36]. Moreover, 80% of the boys and 68% of the girls had Na consumption higher than the recommended age-specific standard dietary target. The Institute of Medicine (IOM) has suggested a recommended upper Na intake amount of 2300 mg/d (100 mmol/d) [1]. In our findings, 41.3% of subjects had Na intake higher than the WHO recommended and 74.2% of children had Na intake higher than the Indonesian recommended age-specific standard dietary target (1500 mg/d). These data are comparable to findings from other studies that revealed the same trends that daily Na intake is still high. A review of population study about salt intake around the world describes most populations including children have mean Na intakes higher than 100 mmol/d and these intakes increase with age [11]. Further analysis showed the Na content of the school food was 606.9 ± 218.9 mg/d. Although it contributed nearly one-third from daily Na intake, the intake exceeded maximum of 30% of school food recommendation (124.8%). The main food sources of Na intake in our study are sweet beverage (74.5%), biscuits (42.55%), crackers (35.5%), and breads (21.28%) (data not shown). These findings are similar with those of previous studies in children from Germany [35], US [37], and Australia [31].

To further understand about high Na intake concern in the school environment, we analyzed factors contributing the nutrient including the role of pocket money. Since food source rich in Na was categorized as unhealthy food and previous studies revealed pocket money is a risk factor for unhealthy eating [38, 39], this study found no association of pocket money with Na intake. Although majority of food sources with Na intake was categorized as unhealthy food and previous studies showed pocket money is a risk factor for unhealthy eating [41, 42], this study found no association of pocket money with Na intake. When analyzing school food, we included all foods consumed by children while at school. Furthermore, in this study, pupils had many access choices to get food at school. Those sources were from pocket money, home-packed lunch, and joined at school meals. Hence, pocket money does not always reflect the nutritional content of food intake by pupils. The school lunch program applied in this school might give a clear explanation. This finding is supported by Li et al.'s [38] study that the association of pocket money with unhealthy food consumption was smaller in schools with restrictions on unhealthy food.

In contrast to Na intake, average daily K intake from urinary analysis in the present study was 16.39 ± 12.57 mmol/d equivalent to 639.15 ± 490.04 mg/d. Only 11.6% and 3.9% of the subjects consumed K intake higher than 1000 mg/d and 2000 mg/d, respectively. Consequently, our analysis indicates that almost all subjects had K intake lower than 1000 mg/d. Furthermore, based on adequate intake, that is, 4500 mg·K/day [1, 40], we did not find any pupils above this level of intake. It is important to note that all subjects had K intake lower than the Indonesian RDA for children aged 9–12 years. Moreover, it is somewhat surprising that urinary K in this study is the lowest than that among children in all previous studies. Other authors have reported higher K intakes than those in our study. A potassium intake of 46.6 ± 23.02 mg/d was reported in Lebanese subjects [21], Grimes et al. [31] in the SONIC study recorded 54 ± 2 mg/dl, and Oliveira et al. [40] reported an average of 43 mmol/d on 8% of children who met the WHO recommendation for K intake. However, our finding is similar to that of two previous studies demonstrating mean K intake in children was approximately 1000mg/d; Kristbjornsdottir et al. [41] showed an average of 1210 mg/d (31 mmol/d) for subjects aged 6 years. A daily K intake of 1000 mg/d (25.64 mmol/d) was reported by Allison et al. [42] in studied children aged 3–5 years. Allison and Walker [42] reported a daily K intake of 1000 mg/d (25.64 mmol/d) in children aged 3–5 years. Based on the WHO suggestion that the recommended K intake is at least 3510 mg/d, our results showed only one subject who met the WHO recommendation for K intake. Moreover, K intake while at school in our study contributed nearly 30% of total daily intake. Thus, our study indicates that almost all children failed to meet the recommended daily K intake.

It is well known that major food sources of K are fruits and vegetables (FV). Really, it is not surprising that K intake in our study is of very low compliance since Indonesian people consume very low fruits and vegetables. This is confirmed by the fact from recent data of Indonesian National Health Survey 2018 that the lack of fruits and vegetables consumption among children more than 5 years increases from 93.5% at 2013 and becomes 95.5% at 2018 [43]. According to the data of Individual Food Consumption Survey 2014, the average consumption of fruits and vegetables of the Indonesian population was 108.8 g/d, which is much lower than that of the World Health Organization (400 g/d); those consumptions were only 81.9 g/d of children aged 5–12 years [44]. The previous studies revealed factors contributing to low fruit and vegetable consumption among children, such as preference, availability/access, and pocket money [45–47].

The preference of FV among students can be analyzed by observing plate waste of lunch meals. Based on observation of their packed lunch and school meals, vegetable is the major food waste, almost all children waste more than 75% of vegetables. The present findings, like those from several population studies, demonstrated that elementary school students wasted a significant amount of these foods; it might indicate that they did not like vegetable items in the menu [45, 46]. Furthermore, the children's preference for FV can be known from observing their home-packed lunch menus. The analysis of home-packed lunch in this study showed that only 4.6% and 15.7% of subjects brought fruits and vegetables, respectively, in the lunch box. Generally, the lunch box was prepared by mothers; however, we did not ask directly about preference of these foods from neither mother nor children, but it might be explained by the previous study that habitual intake of children was mostly influenced by parents' (mother's) food preference [47]. Another factor causing low intake of FV in this study is the limitedness of healthy food in canteen. Observing school canteen, we found that FV items in the menu were very limitedly available at the school canteen. This finding is similar to that of Gabriel et al.'s study that many canteens offered menus of low nutritional value and the items, which were least likely available, were fruits [48]. Related to pocket money, there is no association between pocket money and potassium intake, although we found that they spent their pocket money mostly for buying some sweets, beverages, biscuits, and breads since fruit and vegetable items in the menu were very limitedly available at the school canteen. Finally, in this finding, urinary Na and K correlated significantly (*r*=0.412,  *p* < 0.001), and both were associated with energy of school food and correlated with nutritional status (Table 5). Therefore, it is obvious to consider that the nutritional content of school food is very important since it can contribute to nutritional and health status among schoolchildren.

The limitation of this study is the dietary method used for assessing Na and K intake. Often, dietary Na and K were underestimated or overestimated [19]. Our study reported that potassium intake from recall data was much higher than that of urinary K excretion. Perhaps, these results could be explained by overestimated recorded dietary K because of the food waste and estimation method. It suggests the weakness of our study is that we estimated using household measures of school food rather than food-weighing methods. Conversely, sodium intake from recall data showed underestimation; it was lower than that of urinary Na excretion. A similar finding was also described in studies on Indonesian older women that Na intake from the dietary method was less than that in urine; on the other hand, K intake from the dietary method was greater than that in urine [27]. Another paucity of this study is that we applied spot urines rather than 24 h urinary excretion, although these methods have been validated and performed by a previous study [21].

To the best of our knowledge, this is the first study supplying data on urinary Na and K excretion in Indonesian schoolchildren. In Indonesia, urine has been previously used to assess these nutrients but limited on adults [27], and it has not been yet used especially in children. Focus on school food related to dietary Na and K is still rarely studied by other studies. By evaluating the Na and K component of school foods, we confirmed the importance of these nutrients on the population level particularly to meet nutrient-based standard among schoolchildren. Future research should examine the direct impact of these nutrients on adverse health effects of children such as obesity and hypertension. Furthermore, more studies evaluating all factors that cause excessive Na and deficient K intakes among schoolchildren are needed to support the present findings. Finally, the most important thing is making comprehensive strategies to get healthy diet for children; hence, it is urgent to encourage environmental school setting to get the best way for improving the nutritional intake in schoolchildren by educating pupils, providing healthy school foods such as fruits and vegetables, and of course creating supporting policies.

## 5. Conclusion

In summary, the study reports that the Indonesian schoolchildren aged 9–12 years are categorized by excessive Na intake and very deficient K intake. Since school food has a great contribution to cause this condition, it is vital to encourage the environmental school setting to perform the best way for improving the nutritional intake, particularly Na and K intake among schoolchildren.

## Figures and Tables

**Table 1:** Baseline characteristics of the study participants (*n*=155).

Variable	Frequency or mean ± SD	Percentage
Age (years)	10.15 ± 0.77	
9-10	104	67.1
11-12	51	32.9
Gender		
Boys	80	51.6
Girls	75	48.4
School grade		
4th grade	96	62
5th grade	59	38
Body weight (kg)	39.84 ± 11.84	
Body height (cm)	141.73 ± 7.6	
BMI (kg/m^2^)	19.53 ± 4.4	
Nutritional status (BMI *z*-score)	0.69 ± 1.64	
Underweight	10	6.5
Normal weight	68	43.9
Overweight	40	25.8
Obesity	37	89.67
Pocket money (Rp)	11954.84 ± 5197.83	

**Table 2:** Predicted 24 h urinary Na and K by spot urine excretion stratified by gender.

Variable	Total (*n*=155)	Boys (*n*=80)	Girls (*n*=75)	*p*
Urinary Na/Cr (mmol)	18.16 ± 11.69	17.96 ± 10.01	18.36 ± 13.31	0.833
Urinary K/Cr (mmol)	2.77 ± 1.99	2.81 ± 1.98	2.73 ± 1.98	0.784
P24 h Na (mmol/d)	105.42 ± 66.05	105.72 ± 50.75	105.11 ± 79.55	0.406
P24 h Na (mg/d)	2424.77 ± 1519.09	2431.5 ± 1167.15	2417.58 ± 1829.75	
P24 h K (mmol/d)	16.39 ± 12.57	17.37 ± 13.44	15.34 ± 11.55	0.764
P24 h K (mg/d)	639.15 ± 490.04	677.49 ± 524.32	598.25 ± 450.52	
Urinary Na/K (mmol)	7.71 ± 4.02	7.84 ± 4.48	7.57 ± 3.48	0.963
Urinary Na/K (mg)	4.55 ± 2.37	4.63 ± 2.64	4.47 ± 2.05	

**Table 3:** Estimated/predicted Na and K intake and percentage based on Indonesian RDA (%).

Na intake	Total (155), *n* (%)	Boys (80), *n* (%)	Girls (75), *n* (%)
Estimated Na intake			
<1500 mg/d	40 (25.8)	16 (20)	24 (32)
1500–2300 mg/d	51 (32.9)	30 (37.5)	21 (28)
>2300 mg/d	64 (41.3)	34 (42.5)	30 (40)

Estimated K intake			
<1000 mg	137 (88.4)	66 (82.5)	71 (94.7)
1000–2000 mg	12 (7.7)	9 (11.2)	3 (4)
>2000 mg	6 (3.9)	5 (6.3)	1 (1.3)
≥4500 mg	0	0	0

**Table 4:** The contribution of school food to Na and K daily intake and school standard.

Variable	Full-day food, mean ± SD	School food, mean ± SD	Percentage of school food compared to daily intake	Percentage of school food compared to nutritional school standard
Energy (kcal)	1896.55 ± 276.4	810.9 ± 98.14	42.76	128.7
Sodium (mg)	1820.93 ± 994.06	606.9 ± 218.9	33.33	124.8
Potassium (mg)	1319.82 ± 906.07	393.12 ± 250.9	29.78	24.96

**Table 5:** Factors contributing estimated Na and K intake.

Variables	Estimated Na intake			*p*	Post hoc tests			Estimated K intake			*p*	Post hoc tests		
	<1500 mg/d (*n*=40)	1500–2300 mg/d (*n*=51)	>2300 mg/d (*n*=64)					<500 mg/d (*n*=78)	500–1000 mg/d (*n*=59)	>1000 mg/d (*n*=18)				
Age (years)	10.18 ± 0.59	10 ± 0.8	10.25 ± 0.84	0.219^1^	0.28^2^	0.08^3^	0.63^4^	10.19 ± 0.76	10.05 ± 0.79	10.28 ± 0.75	0.48	0.29	0.28	0.67
Pocket money (Rp)	11800 ± 4479.013.12	12000 ± 6118.82	12015.63 ± 4887.6	0.977	0.86	0.98	0.84	11897.44 ± 5539.91	11661.02 ± 4674.25	13.166.67 ± 5415.12	0.89	0.79	0.28	0.35
BMI *z*-score	−0.07 ± 1.55	0.61 ± 1.52	1.22 ± 1.61	0.001^*∗*^	0.04	0.04	0.00	0.49 ± 1.62	0.65 ± 1.67	1.65 ± 1.39	0.15	0.59	0.02	0.007
Body weight (kg)	34.3 ± 10.75	37.99 ± 9.52	44.78 ± 12.32	0.001^*∗*^	0.12	0.00	0.00	37.59 ± 10.04	39.96 ± 12.06	49.16 ± 14.18	0.02^*∗*^	0.23	0.003	0.000
Energy of full day food (kcal/d)	1879.29 ± 275.49	1929.57 ± 298.81	1881.02 ± 262.29	0.586	0.39	0.35	0.97	1881.07 ± 290.72	1902.64 ± 272.67	1943.69 ± 237.69	0.49	0.65	0.58	0.39
Energy of school food (kcal)	787.02 ± 70.88	808.31 ± 108.51	827.89 ± 102.86	0.117	0.30	0.29	0.04	798.2 ± 91.08	809.44 ± 101.43	870.71 ± 102.96	0.10	0.51	0.02	0.005

^1^ANOVA test; pos hoc test between ^2^groups 1 and 2, ^3^groups 2 and 3, and ^4^groups 1 and 3. ^*∗*^Significant at *p* < 0.05.

## Data Availability

The data used to support the findings of this study are available from the corresponding author upon request and restricted by the ethics of Faculty of Public Health, Universitas Airlangga in order to protect subject privacy. Data Availability statements and articles from Journal of Nutrition and Metabolism provided here are courtesy of Hindawi Limited.
